# Proteomics of adjacent-to-tumor samples uncovers clinically relevant biological events in hepatocellular carcinoma

**DOI:** 10.1093/nsr/nwad167

**Published:** 2023-06-02

**Authors:** Hongwen Zhu, Youpei Lin, Dayun Lu, Shisheng Wang, Yuejia Liu, Liangqing Dong, Qian Meng, Jing Gao, Yuqiu Wang, Nixue Song, Yuying Suo, Li Ding, Pei Wang, Bing Zhang, Daming Gao, Jia Fan, Qiang Gao, Hu Zhou

**Affiliations:** Department of Analytical Chemistry, State Key Laboratory of Drug Research and CAS Key Laboratory of Receptor Research, Shanghai Institute of Materia Medica, Chinese Academy of Sciences, Shanghai 201203, China; Department of Liver Surgery and Transplantation, Liver Cancer Institute, Zhongshan Hospital, and Key Laboratory of Carcinogenesis and Cancer Invasion of Ministry of Education, Fudan University, Shanghai 200032, China; Department of Analytical Chemistry, State Key Laboratory of Drug Research and CAS Key Laboratory of Receptor Research, Shanghai Institute of Materia Medica, Chinese Academy of Sciences, Shanghai 201203, China; School of Chinese Materia Medica, Nanjing University of Chinese Medicine, Nanjing 210023, China; Institutes for Systems Genetics and NHC Key Lab of Transplant Engineering and Immunology, Sichuan Provincial Engineering Laboratory of Pathology in Clinical Application, West China Hospital, Sichuan University, Chengdu 610041, China; School of Chinese Materia Medica, Nanjing University of Chinese Medicine, Nanjing 210023, China; Department of Liver Surgery and Transplantation, Liver Cancer Institute, Zhongshan Hospital, and Key Laboratory of Carcinogenesis and Cancer Invasion of Ministry of Education, Fudan University, Shanghai 200032, China; Department of Analytical Chemistry, State Key Laboratory of Drug Research and CAS Key Laboratory of Receptor Research, Shanghai Institute of Materia Medica, Chinese Academy of Sciences, Shanghai 201203, China; Department of Analytical Chemistry, State Key Laboratory of Drug Research and CAS Key Laboratory of Receptor Research, Shanghai Institute of Materia Medica, Chinese Academy of Sciences, Shanghai 201203, China; Department of Analytical Chemistry, State Key Laboratory of Drug Research and CAS Key Laboratory of Receptor Research, Shanghai Institute of Materia Medica, Chinese Academy of Sciences, Shanghai 201203, China; Department of Analytical Chemistry, State Key Laboratory of Drug Research and CAS Key Laboratory of Receptor Research, Shanghai Institute of Materia Medica, Chinese Academy of Sciences, Shanghai 201203, China; Department of Analytical Chemistry, State Key Laboratory of Drug Research and CAS Key Laboratory of Receptor Research, Shanghai Institute of Materia Medica, Chinese Academy of Sciences, Shanghai 201203, China; University of Chinese Academy of Sciences, Beijing 100049, China; Department of Medicine, McDonnell Genome Institute, Siteman Cancer Center, Washington University, St. Louis, MI 63108, USA; Department of Genetics and Genomic Sciences, Icahn School of Medicine at Mount Sinai, NewYork, NY 10029, USA; Lester and Sue Smith Breast Center, Department of Molecular and Human Genetics, Baylor College of Medicine, One Baylor Plaza, Houston, TX 77030, USA; University of Chinese Academy of Sciences, Beijing 100049, China; State Key Laboratory of Cell Biology, CAS Center for Excellence in Molecular Cell Science, Shanghai Institute of Biochemistry and Cell Biology, Chinese Academy of Sciences, Shanghai 200031, China; Department of Liver Surgery and Transplantation, Liver Cancer Institute, Zhongshan Hospital, and Key Laboratory of Carcinogenesis and Cancer Invasion of Ministry of Education, Fudan University, Shanghai 200032, China; Department of Liver Surgery and Transplantation, Liver Cancer Institute, Zhongshan Hospital, and Key Laboratory of Carcinogenesis and Cancer Invasion of Ministry of Education, Fudan University, Shanghai 200032, China; Department of Analytical Chemistry, State Key Laboratory of Drug Research and CAS Key Laboratory of Receptor Research, Shanghai Institute of Materia Medica, Chinese Academy of Sciences, Shanghai 201203, China; School of Chinese Materia Medica, Nanjing University of Chinese Medicine, Nanjing 210023, China; University of Chinese Academy of Sciences, Beijing 100049, China

**Keywords:** proteomics, hepatocellular carcinoma, normal tissue adjacent to tumor, heterogeneity

## Abstract

Normal adjacent tissues (NATs) of hepatocellular carcinoma (HCC) differ from healthy liver tissues and their heterogeneity may contain biological information associated with disease occurrence and clinical outcome that has yet to be fully evaluated at the proteomic level. This study provides a detailed description of the heterogeneity of NATs and the differences between NATs and healthy livers and revealed that molecular features of tumor subgroups in HCC were partially reflected in their respective NATs. Proteomic data classified HCC NATs into two subtypes (Subtypes 1 and 2), and Subtype 2 was associated with poor prognosis and high-risk recurrence. The pathway and immune features of these two subtypes were characterized. Proteomic differences between the two NAT subtypes and healthy liver tissues were further investigated using data-independent acquisition mass spectrometry, revealing the early molecular alterations associated with the progression from healthy livers to NATs. This study provides a high-quality resource for HCC researchers and clinicians and may significantly expand the knowledge of tumor NATs to eventually benefit clinical practice.

## INTRODUCTION

Primary liver cancer is the third leading cause of cancer-related death globally, with 830 000 deaths in 2020 [1]. Hepatocellular carcinoma (HCC) comprises 75%–85% of liver cancer cases and is of high heterogeneity. Treatment options for HCC include surgery (partial hepatectomy and liver transplant), ablation, embolization therapy, radiation therapy, targeted drug therapy, immunotherapy and chemotherapy. The development of next-generation sequencing and advanced proteomic technologies have seen considerable efforts to perform molecular characterization of HCC to explore potential strategies and drug targets for precision diagnosis and treatment [2–4].

Normal adjacent tissues (NATs), also known as non-tumor liver tissues, are usually considered as a control group in tumor-related studies. A comprehensive analysis of transcriptomes and genome-derived haplotype-specific somatic copy number alterations suggests that the NAT is a unique intermediate state between healthy tissue and tumor and may accumulate oncogenic events [5,6]. HCC in patients is usually accompanied by chronic hepatitis or cirrhosis, and the cirrhotic liver presents a higher mutational burden than that of the healthy liver [7]. In particular, leptin receptor (*LEPR*) somatic mutations are known to accumulate in hepatitis C virus (HCV)-infected cirrhotic liver, disrupting LEPR signaling and creating a higher susceptibility to hepatocarcinogenesis [8]. Gene-expression signatures correlated with survival in HCC NATs have also been discovered using formalin-fixed, paraffin-embedded tissues [9]. All these studies uncovered important biological information from the analysis of NATs, although the comprehensive analysis of NATs at the proteomic level remains less studied. Additionally, the 5-year recurrence rate of HCC at 70% may be mainly explained by the ‘field defect’ of NATs [10]. Commonly used posttreatments of hepatic resection include antiviral agents, traditional Chinese medicine, immunostimulants, Sorafenib and transcatheter arterial chemoembolization. Analysis of NATs may be helpful in developing specific interventions for recurrence prevention. Previously, we performed a proteogenomic analysis of 159 pairs of HCC tumors and NATs to obtain in-depth proteomic data [3]. Given that proteins act as the main regulators of most biological processes and occupy > 95% of currently known pharmacological targets [11], this dataset prompted us to perform a systematic analysis of the characteristics and heterogeneity of HCC NATs to uncover new biological insights at the proteomic level.

Herein, unsupervised clustering was applied to divide HCC NATs into two subtypes that were associated with different prognosis and recurrence (Subtype 1, favorable prognosis and low-risk recurrence; Subtype 2, poor prognosis and high-risk recurrence). Subtype 1 was characterized by high levels of metabolic pathways and Subtype 2 was characterized by high levels of extracellular matrix (ECM) and adhesion-related pathways. Our systematic investigation also revealed clear differences in immune features between these two subtypes. Moreover, a proteomic comparison using healthy liver tissues and the two NAT subtypes and their paired tumors by data-independent acquisition mass spectrometry (DIA-MS) further uncovered early molecular alterations associated with the progression from healthy livers to NATs. Additionally, comparing the healthy–NAT-tumor profiles enabled classification of proteins into four clusters, and the biological characteristics of each cluster were then identified. Overall, this study presents a comprehensive proteomic landscape of NAT heterogeneity in HCC and deciphers its difference from healthy livers. Our study provides a rich resource that sheds new light on HCC-related research and could help develop clinical strategies to improve survival and reduce the risk of recurrence.

## RESULTS

### HCC NATs possess molecular features from their paired tumors

**Figure 1.**
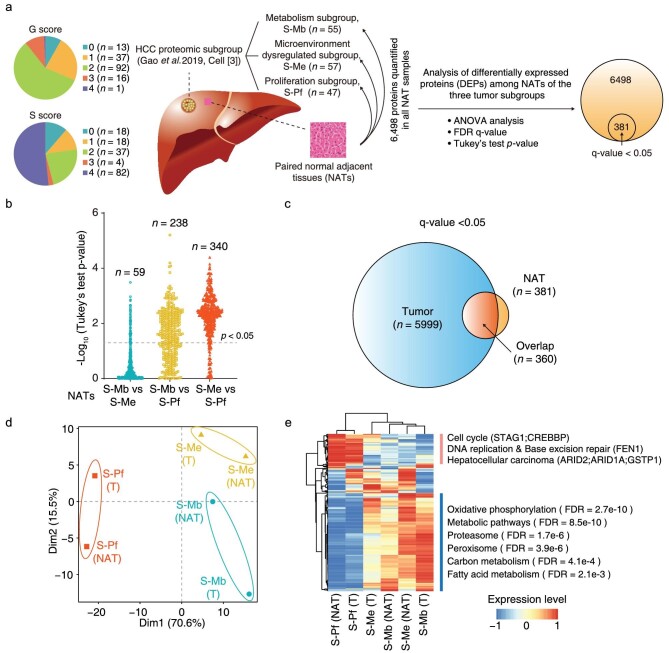
HCC NATs show similar molecular features which are revealed by their paired tumors. a. Analysis of differently expressed proteins (DEPs) among the paired NATs of three HCC proteomic subgroups. Proteins are considered as differently expressed ones by one-way ANOVA analysis followed by adjusting for multiple testing with an FDR q-value < 0.05. Then, the Tukey's test is used for multiple comparisons to analyze the differences between every two subgroups. S-Mb, metabolism subgroup; S-Pf, proliferation subgroup; S-Me, microenvironment dysregulated subgroup. The distributions of patient count with a G score of 0–4 and an S score of 0–4 are shown in the left panel. The schematic diagram of liver tissue was adapted from Gao *et al.* [3]. b. Distributions of negative log10 transformed Tukey's test *p*-value between NATs of every two subgroups. The counts of proteins with *p*-value < 0.05 are shown above. c. An overlap analysis of DEPs among the paired NATs of three HCC proteomic subgroups and DEPs among the three HCC proteomic subgroups. d. Principal component analysis (PCA) of NATs and tumors of the three subgroups using the 360 overlapped DEPs. The median value was used for each NAT or tumor subgroup and scaled at protein level in NATs and tumors, respectively, and then the data were combined for PCA. T, abbreviation for tumor. e. Hierarchical clustering analysis (HCA) of NATs and tumors of the three subgroups using the 360 overlapped DEPs. The representative enriched KEGG pathways and proteins were listed on the right side of the heatmap. All 6498 proteins were used as the background reference list in enrichment analysis.

To understand whether the intrinsic characteristics of HCC tumor subtypes were reflected in NATs at the proteomic level, we performed the following analysis using a previously published proteogenomic dataset of HCC [3]. We acquired an in-depth HCC proteome using isobaric tandem mass tag (TMT)-11 labeling experiments, among which 6498 proteins were quantified across all the 159 paired NATs from the three HCC subgroups, i.e. metabolism subgroup (S-Mb; *n* = 55), microenvironment dysregulated subgroup (S-Me; *n* = 57) and proliferation subgroup (S-Pf; *n* = 47) (Fig. 1a and Table S1). Clinically, in China, the G score (0–4) represents the grading system for inflammation while the S score (0–4) represents the staging system for fibrosis to evaluate the level of chronic hepatitis. The patient counts with a G score of 0–4 were 13, 37, 92, 16 and 1, showing that 89.3% of patients had no or limited inflammation (score = 0, 1 and 2). The patient counts with an S score of 0–4 were 18, 18, 37, 4 and 82, showing that 54.1% of patients had severe liver fibrosis or cirrhosis (score = 3 and 4). The NAT proteome data were adjusted by removing batch effects caused by multiple sets of TMT experiments and then evaluated using hierarchical clustering analysis (HCA) and principal component analysis (PCA) (Figs. S1a and b). A total of 381 proteins were shown to be differently expressed in the paired NATs from the three subgroups by one-way analysis of variance testing (Fig. 1a). After a follow-up multiple comparisons test, the NATs of the S-Me and S-Pf subgroups exhibited the largest difference with 340 differently expressed proteins (DEPs). NATs of the S-Pf and S-Mb subgroups showed a medium difference with 238 DEPs, and those of the S-Mb and S-Me subgroups had the least with 59 DEPs. These comparisons suggested that NATs of the S-Pf subgroup exhibited a different protein expression pattern compared with that of the NATs of the other two subgroups (Fig. 1b).

Among the 381 DEPs, 360 were also differently expressed among the three tumor subgroups which produced 5999 DEPs using a *q*-value < 0.05 (Figs. 1c and S1c). Next, we considered whether the 360 proteins showed a similar changing trend in NATs of the three subgroups to that in the three tumor subgroups. The quantification data of the 360 proteins were first scaled at the protein level in NATs and tumors, respectively, and then the data were combined for PCA and HCA. This revealed that the NAT and tumor of the S-Pf subgroup were clustered together and had similar molecular features (Figs. 1d and e). These features were characterized by lower expression of proteins that were significantly enriched in specific Kyoto Encyclopedia of Genes and Genomes (KEGG) pathways, including oxidative phosphorylation, metabolic pathways, proteasome, peroxisome, carbon metabolism and fatty acid metabolism. Proteins involved in the cell cycle (STAG1, CREBBP), DNA replication and base excision repair (FEN1), and hepatocellular carcinoma (ARID2, ARID1A, GSTP1) also exhibited higher expression in NAT and tumor of the S-Pf

subgroup (Fig. 1e). Thus, the molecular features of the S-Pf subgroup were partially reflected in their respective NATs, suggesting that NATs can predict the biology of specific tumor subtypes.

### The proteome-stratified HCC NATs formed two subtypes associated with prognosis and recurrence

**Figure 2.**
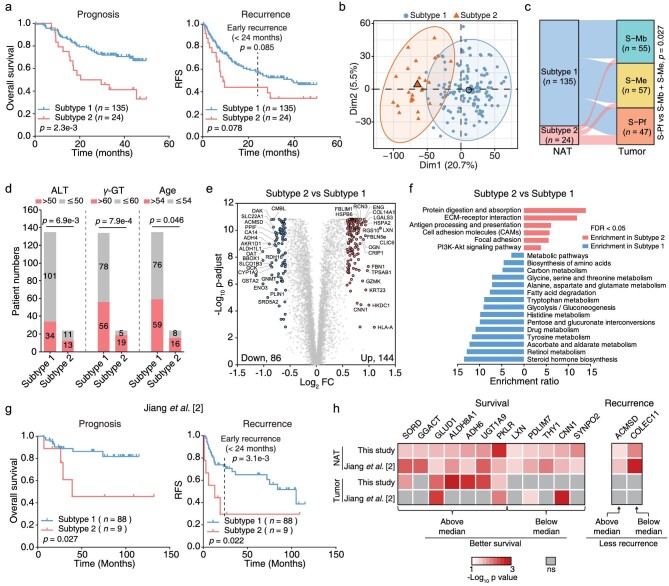
HCC NATs show heterogeneity and can be separated into two subtypes associated with prognosis and recurrence. a. Kaplan–Meier curves of overall survival and cumulative recurrence for the two NAT subtypes. The recurrence plot was marginally significant with *p* = 0.078 (log-rank test). RFS, recurrence-free survival. The dotted line indicates time of 24 months. The statistical difference of early recurrence (<24 months) between two NAT subtypes was calculated by data truncation at 24 months. b. PCA of 159 HCC NATs using all 6498 proteins. Samples of the two NAT subtypes were labeled in different colors. Subtype 1, blue; Subtype 2, red. c. The Sankey diagram shows the matching of paired tumors of the two NAT subtypes in three tumor subgroups. d. Association of age, ALT and γ-GT levels with two NAT subtypes. ALT, aminoleucine transferase (U/L); γ-GT, γ-glutamyl transferase (U/L). (Fisher's exact test). e. The volcano plot shows the differently expressed proteins between two NAT subtypes. The x-axis represents log2 transformed fold changes of Subtype 2 to Subtype 1. The y-axis represents negative log10 transformed Mann–Whitney test *p*-value adjusted by Benjamini and Hochberg (BH) correction. f. Over-represented KEGG pathways in two NAT subtypes. Enrichment analysis was conducted by WebGestalt using all 6498 proteins as the background and statistically significant pathways (FDR < 0.05) were selected for show. g. Differently expressed proteins in (e) were used to perform subtyping using another reported HCC NAT cohort by consensus clustering. Kaplan–Meier curves of overall survival and cumulative recurrence for the resulting two NAT subtypes are shown (log-rank test). The dotted line indicates time of 24 months. h. Differently expressed proteins in (e) were used to calculate the association of protein expression in HCC NATs or tumors with survival and recurrence by log-rank test using median cutoff. Proteins with a significant association (*p*-value < 0.05) in both NAT datasets are shown. The grey blocks represent proteins that are not significant (ns).

The NATs had a lower gene-level mRNA-protein variation correlation (median *rho*, 0.14) than that in HCC tumors (median *rho*, 0.54 [3]) (Fig. S2 and Table S2). Considering that the tumor subgroups significantly differed in survival [3], we chose to explore potential prognosis association in NATs from the proteomic level. To clarify the heterogeneity of HCC NATs, we first used a criterion of median absolute deviation value > 0.3 to filter proteins that were variably expressed in HCC NATs and identified 759 proteins (11.7% of the proteomic dataset). We attempted to stratify the NATs using these proteins. Unsupervised clustering separated the NATs into two subtypes (Subtype 1, *n* = 135; Subtype 2, *n* = 24) (Fig. S3a). Interestingly, these two NAT subtypes significantly differed in overall survival with a poor prognosis in Subtype 2 (log-rank test; *p* = 2.3e-3) (Fig. 2a, left panel). The two subtypes also showed a separated tendency in the recurrence-free survival plot (log-rank test, marginally significant with *p* = 0.078) (Fig. 2a, right panel). It is generally considered that early recurrences before 24 months may come from the primary HCC, whereas recurrence after 24 months probably comes from *de novo* tumors. The early recurrence between the two NAT subtypes was also marginally significant (log-rank test; *p* = 0.085) (Fig. 2a, right panel). Similarly, we used the transcriptome data to perform NAT subtyping, identifying two transcriptomic subtypes with 74 and 85 patients, respectively (Fig. S3b). Compared with the proteomic subtypes, the transcriptomic subtypes showed no differences in prognosis or recurrence, which highlighted the superiority of protein-level molecular subtyping (Fig. S3c). The proteomic Subtype 2 showed a tendency to have a higher occupation in transcriptomic Subtype 2 (Fig. S3d). Furthermore, the proteomic subtypes were authenticated as an independent prognosticator in multivariable analysis (hazard ratio, 2.2; 95% confidence interval, 1.2–4.0; *p* = 1e-2) after considering serum alpha-fetoprotein (AFP) levels (Fig. S3e).

PCA based on all the quantified proteins showed a clear separation between these two NAT subtypes (Fig. 2b). By comparing with the paired tumors, 12 samples of Subtype 2 NATs were mapped to the HCC S-Pf subgroup, showing a higher representation in Subtype 2 NATs (Fishers’ exact test; *p* = 0.027) (Fig. 2c). Patients of Subtype 2 NATs were older (Fishers’ exact test; *p* = 0.046), and association analysis of the clinical information with these two NAT subtypes suggested higher levels of aminoleucine transferase (ALT; >50 U/L; *p* = 6.9e-3) and γ-glutamyl transferase (γ-GT; >60 U/L; *p* = 7.9e-4), (Fig. 2d). These clinical parameters indicated a more injured liver function in Subtype 2. DEPs were filtered under the criteria with fold change > 1.5 and adjusted *p*-value < 0.01, identifying 144 upregulated and 86 downregulated proteins in Subtype 2 compared with their expression in Subtype 1 (Fig. 2e and Table S3). KEGG pathway enrichment analysis suggested that the upregulated DEPs were enriched in pathways in protein digestion and absorption, ECM-receptor interaction, antigen processing and presentation, focal adhesion and PI3K-Akt signaling pathways. Downregulated DEPs were enriched in various metabolic pathways related to normal liver function, including biosynthesis of amino acids, fatty acid degradation, glycolysis/gluconeogenesis, drug metabolism and steroid hormone biosynthesis (Fig. 2f). ECM-receptors played critical roles in the early stages of hepatocarcinogenesis, and the relative changes during the transition from liver fibrosis and steatohepatitis to HCC have been systematically studied in mouse models [12]. The PI3K-Akt signaling pathway has been strongly associated with HCC, and the inhibition of this pathway could be a promising HCC treatment strategy [13].

Since all the patients included in this study were HBV positive, we further analyzed the proteomic differences in HBV-related factors in these two NAT subtypes. The abundance of viral proteins P, C and S were not significantly different (Fig. S4a), although that of HBV receptor SLC10A1 (namely, NCTP) were lower in Subtype 2 NATs (Fig. S4b). As revealed from the transcriptional factor, bile acid synthesis and efflux-related proteins, the Subtype 2 NATs had slightly impaired function of bile acid metabolism with significant *p*-values (Fig. S4b). Furthermore, most proteins in liver-specific metabolic pathways such as gluconeogenesis, detoxication, and ureagenesis-ammonia were significantly attenuated in Subtype 2 NATs, with some specific enzymes (ALDH3B1, GLS and SOAT1) showing an opposing trend (Fig. S4c). These data indicated a global reprogramming of liver-specific metabolism in Subtype 2 NATs.

Next, we tested whether the 230 DEPs could be used as protein features for NAT subtyping in another independent cohort containing proteomic data from 97 HCC NAT samples [2]. A total of 184 proteins overlapped between our dataset and the reported one, and these 184 proteins were then used for consensus clustering. Agreeing with the above results, NATs in this validation cohort could also be divided into two subtypes associated with prognosis (log-rank test; *p* = 0.027) and recurrence (log-rank test; *p* = 0.022) (Fig. 2g). The early recurrences between the two NAT subtypes in the validation cohort were significantly different (log-rank test; *p* = 3.1e-3) (Fig. 2g, right panel). Furthermore, higher expression of seven proteins (SORD, GGACT, GLUD1, ALDH8A1, ADH6, UGT1A9 and PKLR) and lower expression of five proteins (LXN, PDLIM7, THY1, CNN1 and SYNPO2) were associated with improved survival in both NAT datasets. Higher expression of ACMSD and lower expression of COLEC11 were associated with less recurrence (Fig. 2h). In particular, GGACT, LXN, THY1, SYNPO2, ACMSD and COLEC11 showed no survival or recurrence associations from the tumor data, suggesting these as unique biomarkers from NAT. To summarize, using both clinical association and protein-level enrichment analysis, we identified an NAT subtype that seems to have serious hepatic injury and hepatitis.

### The immune features of the two HCC NAT subtypes

**Figure 3.**
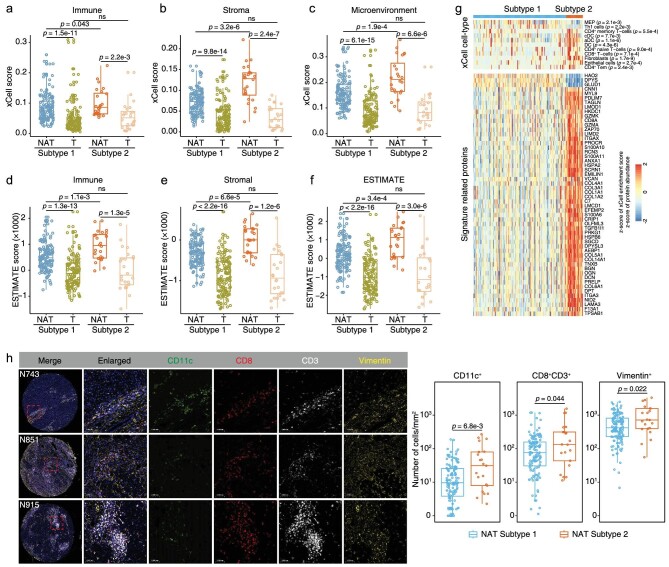
The immune differences of two NAT subtypes. a–c. Boxplots showing immune (a), stroma (b) and microenvironment (c) scores of the two NAT subtypes and their paired tumors calculated by xCell. Differences between NATs and their paired tumors were analyzed by Wilcoxon matched-pairs signed rank test. Differences between two NAT subtypes and differences between the paired tumors of two NAT subtypes were analyzed by Mann–Whitney test. d–f. Boxplots showing immune (d), stromal (e) and ESTIMATE (f) scores of the two NAT subtypes and their paired tumors calculated by ESTIMATE algorithm. g. Heatmap illustrating significantly differential cell type compositions (top panel) and signature related proteins (bottom panel) between two NAT subtypes. The xCell cell-type scores and protein abundances were all normalized by *z*-score. (Mann–Whitney test). h. Left panel: representative multiplexed immunofluorescence staining of sections from Subtype 2 NATs. CD11c, marker of dendritic cells; CD8 and CD3, markers of CD8^+^ T cells; Vimentin, marker of fibroblasts. The patient identification number was labeled in the upper left. Right panel: comparations of the numbers of CD11c^+^, CD8^+^, CD3^+^ T cells and Vimentin^+^ cells between the two NAT subtypes (Mann–Whitney test; Subtype 1, *n* = 128; Subtype 2, *n* = 21).

Subtype 2 NATs have higher levels of inflammatory infiltration as revealed by a higher proportion of patients with clinical G scores of 3 and 4 (Fig. S5a, left panel). The two NAT subtypes did not significantly differ in the level of fibrosis (Fig. S5a, right panel). We investigated the differences in immune infiltration features of these two NAT subtypes using the transcriptome profiles of 159 NATs and paired tumors, and deconvoluted immune, stromal, and microenvironmental cell gene signatures using xCell [14]. Overall, the two NAT subtypes both had higher xCell immune, stroma, and microenvironment scores than those of the paired tumors, indicating higher proportions of immune and stromal cell types in NATs (Fig. 3a–c and Table S4). We had strictly chosen tumor samples with a high tumor purity (81% ± 14% SD) for the previous proteomic analysis [3], which may result in the lower immune infiltration level in tumors. The Subtype 2 NATs had higher proportions of immune and stromal cell types than those in Subtype 1 NATs (Fig. 3a–c), demonstrating a higher level of immune infiltration in NATs from these patients. However, the paired tumor samples of these two NAT subtypes showed no significant differences in these scores (Fig. 3a–c). Another tool for the analysis of the tumor microenvironment, Estimation of STromal and Immune cells in MAlignant Tumors using Expression data (ESTIMATE) [15], also produced similar conclusions as those of xCell (Fig. 3d–f). The purity is defined as the proportion of cancer cells in the tumor tissue or the proportion of normal cells in the NAT tissue, which reflects the features of the tissue microenvironment by considering infiltrating stromal and immune cells. Consequently, the NATs had a lower purity than their paired tumors, and the Subtype 2 NATs had much a lower purity than Subtype 1 NATs, without significant differences in their paired tumors (Fig. S5b).

Next, we analyzed the differences in xCell deconvoluted cell types and the immune signature-related proteins (Fig. 3g). The Subtype 2 NATs had significantly higher proportions of CD4^+^ memory T cells (Mann–Whitney test; *p* = 5.5e-4), conventional dendritic cells (cDC; *p* = 7.7e-3), activated dendritic cells (aDC; *p* = 1.1e-6), dendritic cells (DC; *p* = 4.3e-6), CD4^+^ naive T cells (*p* = 9.0e-4), CD8^+^ T cells (*p* = 7.1e-4), fibroblasts (*p* = 1.7e-9), epithelial cells (*p* = 2.7e-4) and CD4^+^ Tem (*p* = 2.4e-3), and lower proportions of megakaryocyte–erythroid progenitors (*p* = 2.1e-3) and type 1 T-helper cells (Th1; *p* = 2.2e-3) (Fig. 3g, top panel). The higher proportions of DCs, CD8^+^ T cells and fibroblasts were further validated by multiplexed immunofluorescence (Fig. 3h). Type 1 cDC cells could induce inflammatory and a more aggressive behavior in T cells in the lymph nodes responsible for the liver, causing liver damage and liver disease progression [16]. The metabolic activation of intrahepatic CD8^+^ T cells can cause nonalcoholic steatohepatitis and further liver cancer via cross-talk with hepatocytes [17]. Fibroblast aggravation is widely accepted to be derived from activated hepatic stellate cells, which favors tumor survival and is associated with a poor prognosis after curative HCC resection [18,19].

Additionally, we found that the 230 DEPs between the two NAT subtypes were enriched with immune signature-related proteins (*n* = 54), including DPT, PRKG1, SGCD, SCRN1, EMILIN1, LMOD1, HSPB6, C7, TNXB and COL14A1 in fibroblasts; CD8A and GZMK in CD8^+^ T cells; ITGAX, ANXA1 and S100A10 in cDC cells (Fig. 3g, bottom panel), suggesting that the proteomic differences between these two NAT subtypes originated partly from the differences in immune infiltration cell types. The proteome data were also used to perform xCell analysis, and the results were consistent with that from transcriptome data (Figs. S5c and d). The more serious liver inflammation in Subtype 2 NATs was validated by histologic staining (Fig. S5e). Thus, the more activated immune microenvironment in Subtype 2 NATs may contribute to the poorer prognosis and higher recurrence rate.

### Subtype 1 NATs were more similar to healthy liver tissues than subtype 2 NATs

**Figure 4.**
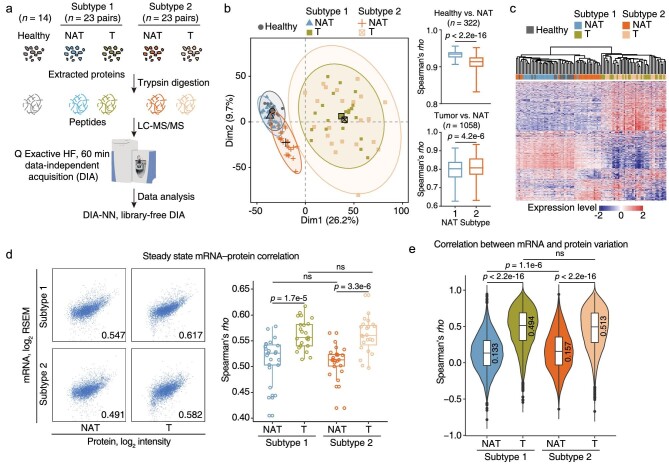
Subtype 1 NATs were similar to healthy liver tissues. a. Workflow of data-independent acquisition (DIA)-based proteomic analysis of healthy livers (*n* = 14), Subtype 1 NATs and paired tumors (*n* = 23), and Subtype 2 NATs and paired tumors (*n* = 23). Proteins were extracted and subjected to trypsin digestion. The MS data were collected on a Q Exactive HF mass spectrometer. The DIA data were analyzed by DIA-NN software in a library-DIA mode. b. Left panel: PCA of healthy livers, Subtype 1 NATs and paired tumors, and Subtype 2 NATs and paired tumors. Right panel: pair-wise Spearman's correlation coefficients between NATs and healthy liver tissues (upper panel; *n* = 322, Mann–Whitney test); pair-wise Spearman's correlation coefficients between NATs and tumor tissues (lower panel; *n* = 1058, Mann–Whitney test). c. HCA of healthy livers, Subtype 1 NATs and paired tumors, and Subtype 2 NATs and paired tumors. d. Correlation of steady state mRNA-protein abundance. Left panel, representative scatter plots of mRNA-protein abundances of two NAT subtypes and their paired tumors. Right panel, the comparison between mRNA-protein correlation coefficients (*rho*) of NATs and that of tumors in each NAT subtype. Differences between NATs and their paired tumors were analyzed by Wilcoxon matched-pairs signed rank test. Differences between two NAT subtypes and differences between the paired tumors of two NAT subtypes were analyzed by Mann–Whitney test. e. Distribution of Spearman's correlation coefficients between mRNA and protein variation in two NAT subtypes and their paired tumors. The median coefficients are labeled above (Kolmogorov–Smirnov test).

To compare the proteomic profiles between the two NAT subtypes and healthy liver, 14 newly collected healthy liver tissues, and 23 NATs from each NAT subtype and their paired tumor tissues randomly selected from the 159-patient cohort in the previous study [3] were assessed using DIA-MS analysis (Fig. 4a and Table S5). The previously used TMT method involved an expensive commercial labeling kit and complicated experimental procedures, whereas the DIA method can avoid these issues and generate permanent digital proteome maps offering rapid, deep, and reproducible retrospective analysis of cellular or tissue samples. The DIA-MS data were analyzed using DIA-NN software with a library-free mode [20]. We identified 6529 proteins in total, among which 5170 proteins were quantified in more than half of the samples. The consistency of chromatographic peak, robustness of protein identification counts (range, 5124 ± 157) and repeatability of quantification correlation in the quality control samples all suggested the high quality of the proteomics data (Fig. S6a–c). The median correlation coefficient across 4661 overlapped proteins between TMT and DIA data (95.6% positive correlations) in 46 NATs was 0.55 (Fig. S6d). The quantification repeatability of the relative changes between the two NAT subtypes was as high as 0.86 (Fig. S6e). These analyses both suggested the satisfactory quantification repeatability between the TMT and DIA methods.

We then analyzed the DIA data from healthy liver tissues, NATs and tumors using PCA and HCA. The paired tumors of the two NAT subtypes were clustered together, showing no obvious separation. Subtype 1 NATs were more similar to healthy liver tissues with higher pair-wise correlation coefficients (Mann–Whitney test; *p* < 2.2e-16), whereas Subtype 2 NATs were much closer to the tumors (Mann–Whitney test; *p* = 4.2e-6) (Fig. 4b and c). mRNA-protein concordance is reported to be associated with tumor subtypes and survival [21]. This prompted us to analyze whether this is the case in HCC NATs. The NATs had smaller steady-state mRNA-protein correlation coefficients compared with their paired tumors and no differences were found between the two NAT subtypes (Fig. 4d). When analyzing the within-gene mRNA-protein correlation, we found a higher median correlation coefficient in Subtype 2 NATs (*rho*, 0.157) than in Subtype 1 NATs (*rho*, 0.133) (Mann–Whitney test; *p* = 1.1e-6) (Fig. 4e). In addition, the median coefficient in tumors is higher than NATs (Fig. 4e), which is consistent with previous reports [21–25].

### The proteomic differences between HCC NATs and healthy livers

**Figure 5.**
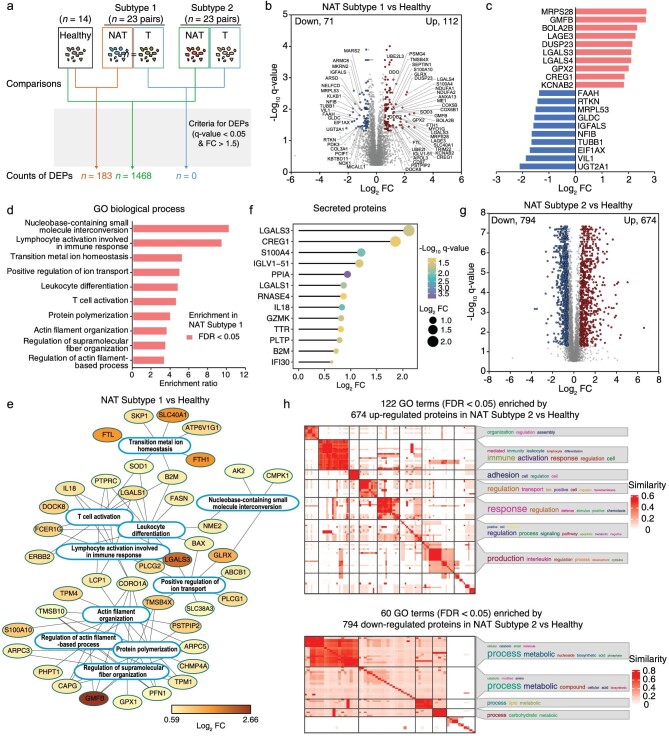
The proteomic differences of two NAT subtypes compared to healthy liver. a. Differently expressed proteins between healthy liver tissues and Subtype 1 NATs, between healthy liver tissues and Subtype 2 NATs, between paired tumors of two NAT subtypes, were calculated using the criteria of FC > 1.5 and *q*-value < 0.05. b. The volcano plot showed the differently expressed proteins between healthy liver tissues and Subtype 1 NATs. The x-axis represented log2 transformed fold changes of Subtype 1 NATs to healthy liver tissues. The y-axis represented negative log10 transformed *q*-value. c. The barplot showed the top ten upregulated proteins and the top ten downregulated proteins. The x-axis represented log2 transformed fold changes of Subtype 1 NATs to healthy liver tissues. d. The significantly enriched biological processes of the upregulated proteins in Subtype 1 NATs compared to healthy liver tissues. The 5170 proteins quantified in more than half of the samples by DIA were input as the background reference list in enrichment analysis. e. The significantly enriched GO biological processes (FDR < 0.05) of the upregulated proteins in Subtype 1 NATs compared to healthy liver tissues. The protein-protein interaction information was extracted from the STRING database. f. The dotplot showed the thirteen upregulated proteins which were annotated as secreted proteins. The negative log10 transformed *q*-values were represented by different colors. The log2 transformed fold changes were represented by different circle sizes. g. The volcano plot showed the differently expressed proteins between healthy liver tissues and Subtype 2 NATs. The x-axis represented log2 transformed fold changes of Subtype 2 NATs to healthy liver tissues. The y-axis represented negative log10 transformed *q*-value. h. The significantly enriched GO terms (FDR < 0.05) in 674 upregulated and 794 downregulated proteins in Subtype 2 NATs compared to healthy livers were displayed by the *binary cut* method for clustering similarity matrices of functional terms.

Except for AFP, no other effective applicable biomarkers are available for the early detection of HCC. A panel of robust biomarkers for early detection would be promising in improving the survival of HCC patients by increasing treatment choices. Differences between Subtype 1 NATs and healthy liver tissues may represent very early molecular events changing from healthy liver to NATs and then to tumors, leading to hepatocarcinogenesis. A total of 183 DEPs were identified between Subtype 1 NATs and healthy liver tissues, including 112 upregulated and 71 downregulated proteins (Fig. 5a and b; Table S6). The top ten upregulated proteins were MRPS28, GMFB, BOLA2B, LAGE3, DUSP23, LGALS3, LGALS4, GPX2, CREG1 and KCNAB2, and the top ten downregulated proteins were UGT2A1, VIL1, EIF1AX, TUBB1, NFIB, IGFALS, GLDC, MRPL53, RTKN and FAAH (Fig. 5c). Biological processes, including actin filament-based process, T-cell activation, positive regulation of ion transport, transition metal ion homeostasis and nucleobase-containing small molecule interconversion, were significantly enriched in the upregulated proteins of Subtype 1 NATs (Fig. 5d and e). No biological processes were highly represented in the downregulated proteins. In addition, 13 upregulated proteins, LGALS3, CREG1, S100A4, IGLV1-51, PPIA, LGALS1, RNASE4, IL18, GZMK, TTR, PLTP, B2M and IFI30, were annotated as secreted proteins, which implies these may be candidates for early biomarkers for fibrosis or HCC diagnosis (Fig. 5f). IL18 was reported to be a useful biomarker in the diagnosis and prognostic prediction in HCC [26,27]. Serum B2M level may be used as a marker for HCV disease progression toward cirrhosis and carcinoma [28].

A total of 1468 DEPs were identified between Subtype 2 NATs and healthy liver tissues, including 674 upregulated and 794 downregulated proteins (Fig. 5g). The DEPs accounted for 28.4% of the total proteins, demonstrating a dramatic proteomic difference in Subtype 2 NATs vs. healthy liver tissues. The upregulated proteins were mainly enriched in biological processes associated with immune activation, adhesion and interleukin production, and the downregulated proteins were over-represented in metabolism-related processes (Fig. 5h), which recovered the molecular features of Subtype 2  vs. Subtype 1 in Fig. 2f. Overall, Subtype 1 NATs were similar to healthy liver tissues, and the DEPs identified between them can be used for biomarker discovery for early liver disease detection. Understanding the NAT subtype-specific characteristics will be helpful in guiding postoperative rehabilitation therapy.

### Dynamic changes in proteins from healthy liver tissues, Subtype 1 NATs and Subtype 2 NATs to tumors

**Figure 6.**
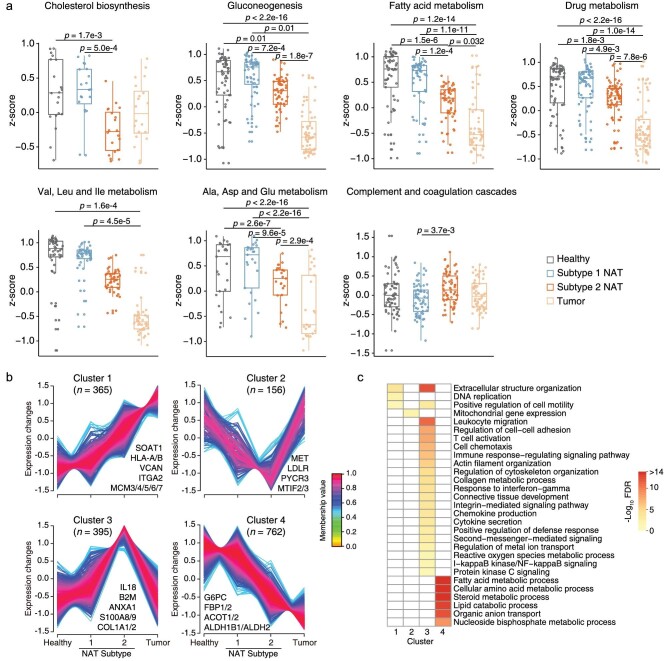
Dynamic changes of proteins and the related biological functions from healthy liver tissues, Subtype 1 NATs, Subtype 2 NATs to tumors. a. The dynamic changes of proteins involved in the core liver functions. The protein abundances were normalized by *z*-score transformation. (Kruskal–Wallis test followed by Dunn's multiple comparisons test). b. The dynamic changes of proteins were divided into four clusters by Mfuzz soft clustering. Only proteins with a membership value > 0.5 were retained. A few representative proteins were labeled to each cluster. c. Representative biological processes were displayed for each cluster. The 5170 proteins of DIA data were input as the background reference list in enrichment analysis.

The Subtypes 1 and 2 NATs had an overlap of 69 upregulated proteins and 48 downregulated proteins, revealing a high proportion of molecular events shared by these two NAT subtypes (Fig. S7a). The sample clustering results (Fig. 4b and c) implied that the dynamic changes of proteins in the shift from healthy liver tissues, Subtype 1 NATs and Subtype 2 NATs to tumors may uncover events associated with hepatocarcinogenesis. First, we examined the changes in proteins involved in key liver functions. The biological processes of gluconeogenesis; fatty acid metabolism; drug metabolism; valine, leucine and isoleucine (V/L/I) metabolism; and alanine, aspartate and glutamate (A/D/E) metabolism showed a decreasing trend, with no significant differences between healthy liver tissues and Subtype 1 NATs (Fig. 6a and Table S7). Subtype 2 NATs seemed to be the intermediate state from healthy livers to tumors in these five processes. The cholesterol biosynthesis-related proteins had much lower levels in Subtype 2 NATs compared with those in healthy liver tissues and Subtype 1 NATs, whereas the levels in Subtype 2 NATs were similar to those in tumors, indicating that cholesterol biosynthesis may be a sensitive process during hepatocarcinogenesis. For the complement and coagulation cascades, Subtype 2 NATs had a higher expression of the related proteins than that in Subtype 1 NATs, which agreed with the inflammation state of Subtype 2 NATs (Fig. 6a). Overall, the Subtype 2 NATs are gradually losing key liver functions.

To systematically analyze the proteins with dynamic changes in the order of healthy liver tissues, Subtype 1 NATs, Subtype 2 NATs and tumors, we clustered all dynamic proteins into four clusters by the Mfuzz soft clustering algorithm, where Cluster 1 proteins (*n* = 365) showed an increasing trend and Cluster 4 proteins (*n* = 762) showed a decreasing trend (Fig. 6b). Cluster 1 proteins were enriched in biological processes, including extracellular structure organization, DNA replication and positive regulation of cell motility. Cluster 4 proteins were enriched in metabolic processes, such as fatty acid metabolism, cellular amino acid metabolism, lipid catabolic process, organic anion transport and nucleoside bisphosphate metabolic process (Fig. 6c). Cluster 2 proteins were gradually downregulated from healthy liver tissues to Subtype 1 NATs, and then to Subtype 2 NATs but restored to similar protein abundance levels as those in healthy liver tissues in tumors. Only one biological process, mitochondrial gene expression, was significantly enriched in Cluster 2 (Fig. 6b and c). The trend of Cluster 3 proteins opposed that of Cluster 2 proteins, with enrichment of processes, such as extracellular structure organization, leukocyte migration, regulation of cell-cell adhesion, T-cell activation, cell chemotaxis, immune response-regulating signaling pathway and regulation of cytoskeleton organization (Fig. 6b and c).

Notably, even though metabolic pathways were widely downregulated in HCC tumors, several specific metabolism-related proteins showed an upregulated tendency. Sterol O-acyltransferase 1 (SOAT1) was involved in cholesterol metabolism and showed an increasing trend as a member of Cluster 1, and has been considered a potential therapeutic target of early-stage HCC [2]. In this context, we aimed to find metabolism-related proteins with an increasing profile from healthy liver tissues, Subtype 1 NATs and Subtype 2 NATs to tumors. Combined with prognosis information from both the Human Protein Atlas and our previous dataset [3], nine proteins, including PAFAH1B3, GPD1L, LPCAT1, DCK, POLD1, ACOT7, SRM, DNMT1 and G6PD, may be involved in hepatocarcinogenesis (Fig. S7b). Interestingly, PAFAH1B3, LPCAT1, DCK, ACOT7 and G6PD have been reported to be related to HCC progression [29–33]. These proteins may be considered as potential drug targets for the treatment of HCC. To summarize, we identified a cluster of proteins (Cluster 1; *n* = 365) showing an increasing trend along the healthy–NAT-tumor profiles, which may be involved in the tumorigenesis of HCC but this needs further functional validation.

## DISCUSSION

This study performed a systematic analysis of the molecular heterogeneity in HCC NATs at the proteomic level using our previously reported dataset. We revealed that the molecular features of tumors can be found from their paired NATs. An NAT subtype of patients with poorer prognosis and easier relapse was identified using unsupervised clustering analysis, showing higher levels of inflammatory infiltration. Furthermore, the proteomic differences among HCC NATs, tumors and healthy liver tissues were compared via DIA-MS analysis, demonstrating that Subtype 1 NATs were more similar to healthy liver tissues than Subtype 2 NATs and that NATs had smaller cross-patient correlation coefficients between mRNA and protein variation compared with their paired tumors. Biological events associated with clinical outcome and disease occurrence were explored and may assist in identifying diagnostic biomarkers, developing therapy strategies and uncovering new tumor promoters.

With the advancement of MS technology, proteomics has been widely applied in large-scale cohort studies for clinical tumor samples. Even though it cannot achieve the sensitivity and depth found in next-generation sequencing, this technology can provide biological information in a new dimension and act as an important supplement to genomic and transcriptomic analyses. The intrinsic characteristics of tumor subtypes can be reflected in the paired NATs, which was demonstrated in breast cancers using transcriptome data [34]. Herein, we analyzed whether proteomic differences were present among NATs of the previously defined three tumor subgroups. NATs of the S-Pf subgroup clearly differed from NATs of the S-Mb and S-Me subgroups and showed molecular features of the paired tumor subgroup. This finding inspired us to explore the heterogeneity in NATs themselves. Proteogenomic characterization of many tumor types has demonstrated the importance and superiority of the proteome in uncovering novel biological insights and therapeutic opportunities [3,22,24,35–45]. In this study, the proteomic data separated the HCC NAT samples into two subtypes by unsupervised clustering using the most variably expressed proteins, identifying two NAT subtypes associated with prognosis and recurrence, whereas no differences were found when using the transcriptomic data.

Molecular subtyping seeks to uncover the heterogeneity of tumors or NATs by classifying the tissues into homogeneous groups that are associated with distinct molecular features, clinical outcomes or therapeutic options. Even though NATs have relatively lower heterogeneity compared with that of tumors, the NATs themselves may contain important biological information. We believe that different biological features should be clarified for each subtype, which may highlight the potential significance of molecular subtyping. Two previously published reports have clustered the HCC NATs into three molecular subgroups at the proteomic level [46,47]. Gu *et al.* performed the consensus clustering using all the identified proteins, and while not giving a description of molecular features for each subtype, did show that several proteins with differences in risk indices were specific to molecular subtypes. Most proteins exhibited similar levels between S-I and S-II subtypes, but apparently different from those in the S-III subtype [46]. Liao *et al.* also divided NATs of their patients into three subtypes, identifying that S1 and S2 patients had similar expression patterns. The S1 and S2 samples demonstrated similar enrichment for immune cell types with only minimal heterogeneity found in the metabolic activity of the three subtypes [47]. To summarize, even though these two reports divided the HCC NATs into three subtypes based on the proteome expression pattern, two of these seemed to be highly similar with no apparently different molecular features. In comparison, the two NAT subtypes identified in our analysis had significantly different proteomic patterns, molecular features and immune infiltration landscapes. The NAT Subtype 1 was highlighted by metabolism-related pathways, and NAT Subtype 2 had a more activated immune microenvironment. Importantly, our subtyping criteria were validated using another independent cohort containing high-quality proteome data of 97 HCC NAT samples.

HCC NATs exhibit hepatic fibrosis or hepatitis and are not regarded as normal liver tissues, so we performed a DIA-MS experiment to compare the proteomic differences among NATs, tumors and healthy livers. This revealed that Subtype 1 NATs were close to healthy livers while Subtype 2 NATs considerably differed from healthy livers, which indicated that care is required when using NATs as controls in tumor-related studies. Both NAT subtypes have much lower cross-patient mRNA-protein correlation coefficients. The correlation analysis between mRNA and protein variation consisted of studying how the variation of each transcript and protein originating from the same gene correlated across all samples. This provided information about whether changes in mRNA levels produced changes in abundance of the corresponding proteins. Tumors are reported to have higher within-gene mRNA-protein correlation coefficients compared with that of NATs in similar studies [21–25]. The differences in mRNA-protein correlation between tumors and NATs can be attributed to several biological factors, which include translation rates that vary across proteins, conditions and cell types, highly variable half-lives for both proteins and mRNAs, and posttranslational modifications that can alter protein stability and degradation [48–50]. However, this may be due to the different variability of mRNA or protein data between NATs and tumors. The intrinsic variability in mRNA expression is low among NATs because they are highly homogeneous compared with the heterogeneous tumors. The correlation coefficient is sensitive to the range of observations [51]. In NATs, mRNAs do not vary across samples and produce a low correlation with their corresponding proteins because the variation is essential to observe the correlation. Tumor mRNA is highly heterogeneous and contributes to the variable protein expression, producing a higher gene-level mRNA-protein correlation.

The development of primary tumors is highly complex and may be caused by a variety of genetic, chemical and physical factors. The ‘field cancerization’ theory implies an evolutionary process of carcinogenesis whereby genetic alterations are acquired step-wise, leaving the NAT tissue in an intermediate, pre-neoplastic state [52]. Another viewpoint is that the tumor has an active role in shaping the microenvironment in the adjacent tissue [5]. Regardless of these different opinions about primary tumor development, wide communication exists between tumors and the surrounding host tissues. The NATs are critical for recurrence-related studies in HCC. In particular, protein changes in NAT Subtype 1 compared with healthy livers may contain factors that have functions in changing the tumor microenvironment and promoting tumor recurrence. The upregulated proteins in Subtype 1 NATs compared with those in healthy livers were enriched with immune-related biological processes, including T-cell activation, leukocyte differentiation and lymphocyte activation, suggesting that these act as contributing factors that may help modulate the tumor microenvironment (TME), which may be a signal for tumor initiation. LGALS3 functions in the organization of the TME and is known to be involved in HCC bone-metastasis [53,54]. LGALS1 played a crucial role in HCC-associated fibroblasts that orchestrate an inflammatory cancer stem-like cell niche and reprogram the inflammatory tumor microenvironment, thus supporting tumor progression [55]. IL18 exerts inflammation-dependent tumor-suppressive effects largely by promoting the differentiation, activity and survival of tumor-infiltrating T cells. Patients with the highest difference in IL18 expression between tumor and nontumor tissues had the poorest prognosis [56]. SLC40A1 is the major iron transporter that controls dietary iron uptake, iron recycling by macrophages and release of iron stores in hepatocytes. SLC40A1 was highly expressed in tumor-associated macrophages and promoted proinflammatory cytokines in TME of HCC [57]. The expression levels of ferritin subunits FTL and FTH1 were positively correlated with tumor infiltration by tumor-associated macrophages and T regulatory cells in most solid tumors, suggesting an important role in regulating tumor immunity [58]. Some proteins were inferred to be involved in TME remodeling via bioinformatics calculation. For example, the expression of GMFB was significantly and positively correlated with macrophage M2 cells and mast resting cell infiltration levels [59]. ARPC5 expression was positively correlated with the tumor microenvironment scores, immune-infiltrating cells and immune checkpoint–related genes in most cancers [60]. FCER1G is a TME-related prognostic signature for HCC [61]. Further experimental investigation needs to be conducted to verify the function of these proteins in regulating TME.

The study of molecular heterogeneity in HCC NATs enables potential basic and clinical translation applications. Protein features differentiating NATs of the S-Pf subgroup from NATs of the other subgroups or stratifying different NAT subtypes may contribute to developing potential clinical prevention strategies to prolong survival and suppress recurrence in these patients. Biomarkers derived from NATs may assist in defining potential targets for early detection, diagnosis or therapeutic intervention. Using our dataset and another high-quality dataset, we identified proteins that showed clinical prognosis and recurrence associations, of which GGACT, LXN, THY1, SYNPO2, ACMSD and COLEC11 were unique biomarkers identified from NAT that could not be revealed using tumor-based analysis. Additionally, DIA analysis identified upregulated expression of 13 secreted proteins in NAT Subtype 1 compared with their expression in healthy livers, enabling possible early detection of HCC through serum, which is also a limitation of this study because validation for these potential biomarkers in a large cohort of serum samples was lacking. This study identified promising candidates for HCC-related basic research. By clustering proteins from healthy liver tissues, Subtype 1 NATs, and Subtype 2 NATs to tumors, we identified 365 proteins that have an increasing expression profile, suggesting that their high expression level may promote cancer development. The molecular function and prognosis association were further inspected, identifying nine metabolism-related proteins with unfavorable prognosis. As metabolic reprogramming is emerging as an important characteristic of cancer [62], potential strategies targeting these proteins may provide novel clues for HCC treatment.

The study of NAT proteomic profiling may help develop interventions for recurrence prevention in future clinical applications. Hughes *et al.* suggested that the selective targeting of relapse-promoting, perivascular tumor-associated macrophages could delay the relapse of both primary and metastatic tumors in patients after chemotherapy, thereby extending their relapse-free survival [63]. For patients with NAT Subtype 2 and a higher risk for recurrence, targeting several types of immune cells or inhibiting the globally activated immune status may be useful in combating recurrence. Additionally, rational combinations of microenvironment-targeting therapies with immune checkpoint inhibitors or cellular therapy will comprise the next generation of immune-based approaches to cancer treatment [64]. Therefore, knowledge of the immune landscape in NATs may help with treating cancer in the future of HCC-related immunotherapy.

Aran *et al.* found that a pan-cancer mechanism of proinflammatory signals from the tumor stimulates an inflammatory response in the adjacent endothelium from the transcriptome analysis [5]. Considering the interesting findings from the proteomic data of HCC, such as the existence of an immune activated subtype and identification of early molecular events in NATs compared with healthy tissues, it should be essential for researchers in the future to study the heterogeneity of NATs in other tumor types from the pan-cancer perspective.

In summary, this study provided a comprehensive proteomics analysis of HCC NATs and determined the proteomic difference between HCC NATs and healthy livers. The high-quality proteomic data generated in this study combined with the data provided in our previous reports [3,35,65,66] are important resources for proteomic and clinical research in the study of liver cancer.

## MATERIALS AND METHODS

### Clinical sample acquisition

The acquisition of tumor and NAT samples has been described previously [3]. The 159 HCC patients underwent primary curative resection from June 2010 to December 2014 at Zhongshan Hospital and received no prior anticancer treatments. Tissue samples were collected within 30 min after operation and snap-frozen in liquid nitrogen. Additionally, 14 healthy liver samples were collected from patients who were diagnosed with hepatic hemangioma (*n* = 13) or focal nodular hyperplasia (*n* = 1) when undergoing surgery at Zhongshan Hospital. The healthy liver tissues were checked by H&E staining. The study was approved by the Research Ethics Committee of Zhongshan Hospital (B2017-060), and written informed consent was obtained from each patient.

### Quantification and statistical analysis

Quantification methods and statistical analysis methods were mainly described and referenced in the respective method details or figure legends. In short, for comparations between two groups of samples, differences between paired samples were analyzed by Wilcoxon matched-pairs signed rank test and differences between non-paired samples were analyzed by Mann–Whitney test. For comparations among more than two groups of samples, Kruskal–Wallis test or one-way ANOVA analysis followed by Dunn's multiple comparisons test was used. For comparations between the distributions of two groups, Kolmogorov–Smirnov test was used. For prognosis analysis, log-rank test was used. For association analysis, Fisher's exact test was used. For boxplots, mean ± standard error (SD) was presented.

## Supplementary Material

nwad167_Supplemental_Files

## Data Availability

The DIA-MS data produced in this study have been deposited to the ProteomeXchange Consortium (http://proteomecentral.proteomexchange.org) via the iProX partner repository [67] with the dataset identifier PXD036048. The transcriptome and proteome data produced in the previous study [3] can be acquired through the NODE platform (https://www.biosino.org/node/project/detail/OEP000321) or the website of CPTAC (https://proteomic.datacommons.cancer.gov/pdc/study/PDC000198).
