# Supplementary material for: Proteomics of adjacent-to-tumor samples uncovers clinically relevant biological events in hepatocellular carcinoma
Source: Natl Sci Rev. 2023 Jun 2;10(8):nwad167. doi: 10.1093/nsr/nwad167 (PMC10416816; doi:10.1093/nsr/nwad167)
Supplement: nwad167_Supplemental_Files [file nwad167_supplemental_files.zip › Supplemental material.docx]

**Methods**

**Preprocessing of the previously published TMT-based proteomic data**

In the previously published dataset, we identified 10783 proteins using 33 sets of TMT-11 plex experiment across 165 pairs of HCC tumor and NAT samples, among which 159 pairs were eventually used for the proteogenomics analysis. A total of 6498 proteins were quantified in all 159 NAT samples. Before performing the downstream analysis, we applied batch correction on the NAT proteomic data using an R tool, ComBat, in “sva” package to remove batch effects brought by the technical differences among different TMT batches. This step was necessary for analysis in this report, because the biological differences may be greatly affected by technical differences especially for the NAT samples with low heterogeneity. PCA and HCA were used to evaluate the effects after batch correction. The ANOVA analysis was used to find DEPs among NATs of the three tumor subgroups and p-values were adjusted by FDR (q-value). Tukey's test for post-hoc analysis was used to compare the differences between NATs of every two tumor subgroups.

**GO biological process and KEGG pathway enrichment analysis**

The enrichment analysis was performed using WebGestalt tool (<http://www.webgestalt.org/>) [1]. The over-representation analysis (ORA) method and BH-based multiple test adjustment were used. The *binary cut* method was used for clustering similarity matrices of functional terms and the R package *simplifyEnrichment* provided functionalities for visualizing, summarizing and comparing the clusterings [2]. The network consisting of the significantly enriched biological processes and the related up-regulated proteins was constructed in Cytoscape [3].

**Prognosis analysis and multivariate Cox regression analysis**

Kaplan-Meier plots (Log-rank test) were used to describe the overall survival or RFS in GraphPad Prism 7.0. To demonstrate the proteome subtypes of NATs as an independent prognosticator, univariate Cox regression analysis was firstly performed for each clinical factor (gender, liver cirrhosis, AFP level, TB level, ALT level, γ-GT level, age and NAT subtype) and the significant factors (AFP level and NAT subtype) were used for the following multivariate Cox regression analysis in R software.

**Correlation analysis**

Spearman correlation coefficient (*rho*) was applied to measure the correlation between mRNA and protein variation, the correlation between mRNA and protein in steady state and the correlation between DIA and TMT quantification. The p-value corresponding to the correlation analysis was computed and adjusted by FDR correction, with an adjusted P value < 0.01 determined to be significantly positive.

**Association analysis**

Association of age, ALT and γ-GT levels with two NAT subtypes was tested by Fisher’s exact test. Association of the proteome subtypes with the transcriptome subtypes was tested by Fisher’s exact test.

**Consensus clustering for proteomic and transcriptomic data**

The most varied proteins (median absolute deviation, MAD >0.3, n=759) of the NAT proteome data (~12%) were used to perform K-means consensus clustering to identify subtypes using ConsensusClusterPlus R package[4], with the following detail settings used for clustering: number of repetitions = 1,000 bootstraps; pItem = 0.8 (resampling 80% of any sample); pFeature = 1 (resampling 100% of any protein); and k-means clustering with up to 8 clusters. The number of clustering was determined by considering the average pairwise consensus matrix within consensus clusters, the consensus cumulative distribution function (CDF) plot and the delta plot of the relative change in the area under the CDF. For the transcriptomic data, the most varied 2000 genes of NAT transcriptome data (~10%) were used to perform K-means consensus clustering to identify subtypes and the parameters were set as above.

**Processing of another published proteomic dataset (Jiang et al. 2019, Nature)**

The previously reported proteomic data of HCC NATs (n=98) was provided using label free quantification with iBAQ intensities[5]. The data was subjected to median normalization and then missing values were imputed by the minimum value in each sample. A total of 184 proteins are overlapped with the DEPs in our data and used for sample subgrouping by consensus clustering. According to the consensus matrix heatmap, consensus CDF plot and delta area plot, 97 NATs were separated into two subtypes (Subtype 1, n=88; Subtype 2, n=9). Specially, one sample (L035P) was shown to be atypical in the PCA analysis for tumor and NAT samples and was eventually deleted for the following subtyping and prognosis analysis.

**Immune infiltration related analysis**

The immune infiltration related analysis was performed using xCell [6] and ESTIMATE [7]. The webtool “<https://xcell.ucsf.edu/>” was used for “xCell” analysis and the R package “estimate” was used for ESTIMATE analysis. The transcriptome data (log_2_ transformed upper-quartile normalized RSEM counts) was directly uploaded for the analysis. For proteomic data based xCell analysis, to ensure enough protein counts used for input, the dataset containing 9042 proteins quantified in at least half NAT samples were used and missing values were imputed using DreamAI algorithm [8]. The proteomic data was transformed by z-score normalization and then used for xCell analysis.

**Multiplexed Immunofluorescence**

Multiplexed Immunofluorescence was performed as we previously described [9]. The Tissue MicroArray (TMA) containing 155 NATs originated from this cohort was used, which was constructed from our previous paper [10]. Briefly, the TMA was melted at 60 ℃ in an oven for 2 hours, deparaffinized with xylene, and rehydrated with different concentrations of alcohol. The first round of antigen retrieval was performed with preheated EDTA buffer (MVS-0080, MXB) for 12 minutes in a pressure cooker and subsequent rounds were performed with preheated citrate buffer (MVS-0101, MXB) for 10 minutes in a microwave to remove unbound Abs-dye complex. Endogenous peroxidase and nonspecific binding sites were blocked with blocking solution (YFH4001, YIFAN Biological) and goat serum (AR0009, BOSTER) for 20 minutes each, respectively. Then the section was incubated with primary Abs at 4 ℃ overnight and followed by secondary HRP-conjugated polymers (GK500710, MXB) at room temperature for 30 minutes and different fluorophores for 10 minutes in the absence of light. The staining regimen was as follows: anti-CD11c (ab52632, Abcam)/CF 488A, anti-CD3 (ab16669, Abcam)/AF647, anti-vimentin (ab92547, Abcam)/Cyanine 3, anti-CD8 (ab17147, Abcam)/ AF594. The cell nucleus was stained with DAPI (Sigma-Aldrich) and the slide was left to dry flat for 30 minutes and stored at 4 ℃ before scanning. The scanning was performed using PANNORAMIC SCAN II (3DHISTECH Ltd.) and the Visiopharm Software 2023.01 (Visiopharm A/S, Hoersholm,Denmark) was used for TMA data analysis. After evaluation of the staining quality, a total of 149 NATs in the TMA were used for the statistical analysis (Subtype 1, n = 128; Subtype 2, n = 21).

**Peptides preparation and DIA-MS experiment**

Healthy liver tissues were subjected to frozen pulverization and protein extraction as described previously [10]. The proteins were digested by SP3 method [11]. The resulted peptides were purified using C18 stage-tips and quantified by NanoDrop 2000 (ThermoFisher Scientific). For each sample, ~1 μg peptides were subjected to LC-MS/MS analysis.

For DIA-MS analysis, the peptides were resolved using 0.1% formic acid and separated using a home-made micro-tip C18 column (75 mm × 200 mm) packed with ReproSil-Pur C18-AQ, 3.0 mm resin (Dr. Maisch GmbH, Germany) on a nanoflow HPLC Easy-nLC 1000 system (Thermo Fisher Scientific), using a 60 min LC gradient at 300 nL/min. Buffer A consisted of 0.1% (v/v) formic acid in H_2_O and Buffer B consisted of 0.1% (v/v) formic acid in acetonitrile. The gradient was set as follows: 1%–4% B in 1 min; 4%–26% B in 47 min; 26%–32% B in 5 min; 32%–90% B in 2 min; 90% B in 5 min. Proteomic analyses were performed on a Q Exactive HF mass spectrometer (Thermo Fisher Scientific). The spray voltage was set at 2,300 V in positive ion mode and the ion transfer tube temperature was set at 300℃. Data-independent acquisition was performed using Xcalibur software in profile spectrum data type. The MS1 full scan was set at a resolution of 120,000 @ m/z 200, AGC target 3e6 and maximum IT 100 ms by orbitrap mass analyzer (340-1510 m/z), followed by 40 DIA isolation windows with variable width which were decided by the searching result of a preliminary DDA experiment. The isolation windows were set with 1 Da overlap as follows: 1 loop count of 26 m/z with central m/z at 363; 3 loop counts of 16 m/z with central m/z at 383, 398, 413; 16 loop counts of 12 m/z with central m/z at 426, 437, 448, 459, 470, 481, 492, 503, 514, 525, 536, 547, 558, 569, 580, 591; 9 loop counts of 14 m/z with central m/z at 603, 616, 629, 642, 655, 668, 681, 694, 707; 4 loop counts of 21 m/z with central m/z at 723.5, 743.5, 763.5, 783.5; 3 loop counts of 26 m/z with central m/z at 806, 831, 856; 1 loop count of 36 m/z with central m/z at 886; 1 loop count of 51 m/z with central m/z at 928.5; 1 loop count of 81 m/z with central m/z at 993.5; 1 loop count of 468 m/z with central m/z at 1267. The MS2 scans generated by HCD fragmentation at a resolution of 15,000 @ m/z 200, AGC target 1e6 and maximum IT auto. The fixed first mass of MS2 spectrum was set 100.0 m/z. The normalized collision energy (NCE) was set at NCE 27%. The DIA-NN 1.7.16 was used for spectral library-free DIA searching against the human Swiss-Prot database containing 21,155 sequences (downloaded in September, 2019) [12]. The default searching parameters were used.

For the searching result produced by DIA-NN, the file “report.unique_genes_matrix.tsv” was used for the following analysis. A total of 5170 proteins were quantified in more than half samples. The proteomic data were normalized by median values and the missing values were imputed by the “replace missing values from normal distribution” function in Perseus software [13].

**Core liver function related proteins**

The protein lists involved in the core liver metabolism functions were retrieved from a previously published paper[14]. The proteins involved in complement and coagulation cascades were retrieved from KEGG (<https://www.genome.jp/kegg/>). The protein abundances were normalized by z-score transformation.

**Mfuzz soft clustering**

The mfuzz clustering was performed using an R package “Mfuzz”. Only proteins with of a membership value >0.5 were regarded as the core proteins of the clusters.

**References**

1. Liao Y, Wang J, Jaehnig EJ *et al.* WebGestalt 2019: gene set analysis toolkit with revamped UIs and APIs. *Nucleic Acids Res* 2019;**47**:W199–205.

2. Gu Z, Hübschmann D. Simplify enrichment: A bioconductor package for clustering and visualizing functional enrichment results. *Genomics Proteomics Bioinformatics* 2022, DOI: 10.1016/j.gpb.2022.04.008.

3. Shannon P, Markiel A, Ozier O *et al.* Cytoscape: a software environment for integrated models of biomolecular interaction networks. *Genome Res* 2003;**13**:2498–504.

4. Wilkerson MD, Hayes DN. ConsensusClusterPlus: a class discovery tool with confidence assessments and item tracking. *Bioinformatics* 2010;**26**:1572–3.

5. Jiang Y, Sun A, Zhao Y *et al.* Proteomics identifies new therapeutic targets of early-stage hepatocellular carcinoma. *Nature* 2019;**567**:257–61.

6. Aran D, Hu Z, Butte AJ. xCell: digitally portraying the tissue cellular heterogeneity landscape. *Genome Biol* 2017;**18**:220.

7. Yoshihara K, Shahmoradgoli M, Martínez E *et al.* Inferring tumour purity and stromal and immune cell admixture from expression data. *Nat Commun* 2013;**4**:2612.

8. Ma W, Kim S, Chowdhury S *et al.* DreamAI: algorithm for the imputation of proteomics data. *bioRxiv* 2021:2020.07.21.214205.

9. Lin Y, Peng L, Dong L *et al.* Geospatial Immune Heterogeneity Reflects the Diverse Tumor–Immune Interactions in Intrahepatic Cholangiocarcinoma. *Cancer Discov* 2022;**12**:2350–71.

10. Gao Q, Zhu H, Dong L *et al.* Integrated Proteogenomic Characterization of HBV-Related Hepatocellular Carcinoma. *Cell* 2019;**179**:561–77.

11. Hughes CS, Moggridge S, Müller T *et al.* Single-pot, solid-phase-enhanced sample preparation for proteomics experiments. *Nat Protoc* 2019;**14**:68–85.

12. Demichev V, Messner CB, Vernardis SI *et al.* DIA-NN: neural networks and interference correction enable deep proteome coverage in high throughput. *Nat Methods* 2020;**17**:41–4.

13. Tyanova S, Temu T, Sinitcyn P *et al.* The Perseus computational platform for comprehensive analysis of (prote)omics data. *Nat Methods* 2016;**13**:731–40.

14. Gong Y, Ji P, Yang Y-S *et al.* Metabolic-Pathway-Based Subtyping of Triple-Negative Breast Cancer Reveals Potential Therapeutic Targets. *Cell Metab* 2021;**33**:51–64.


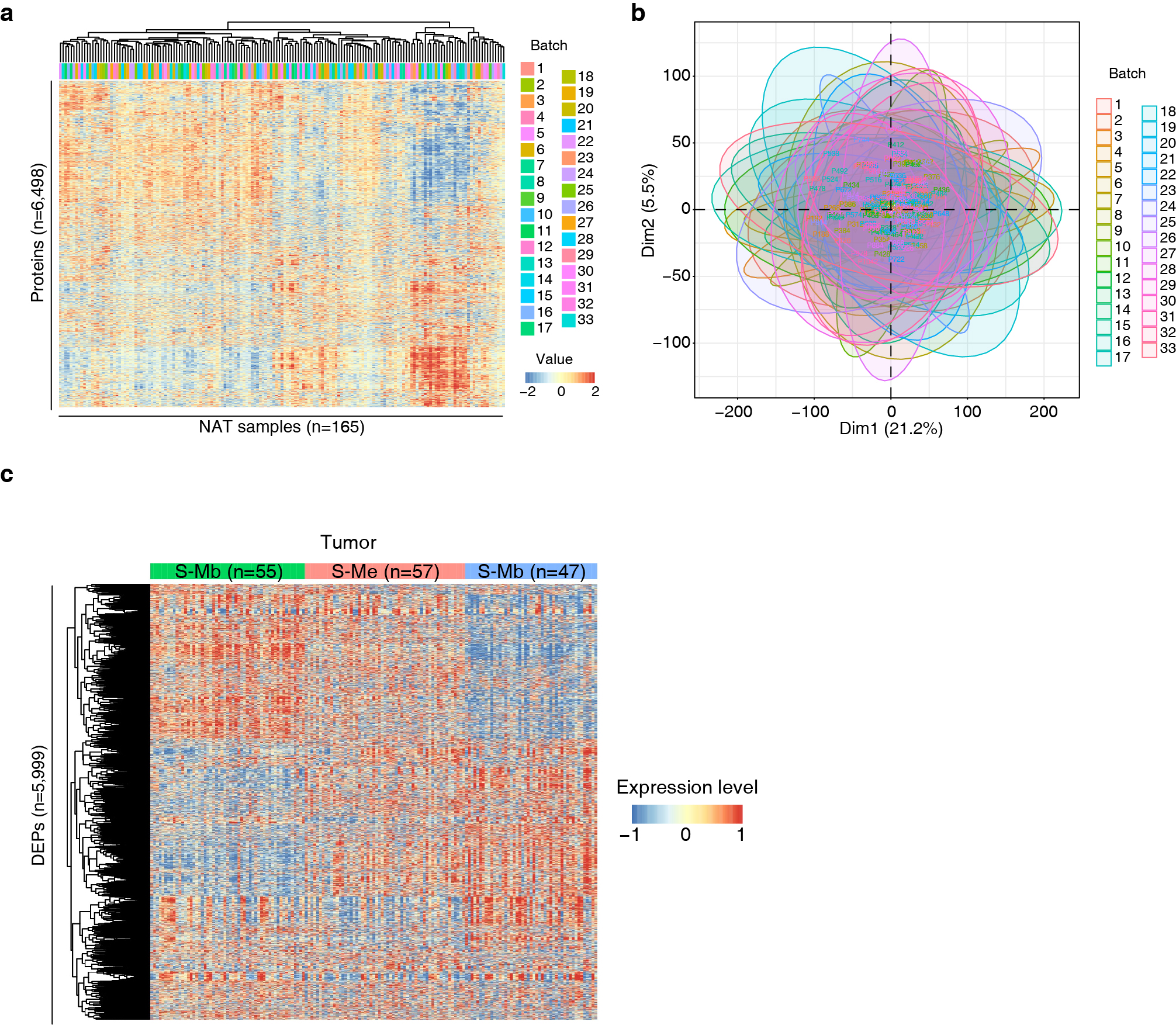


**Figure S1. Batch correction in HCC NAT data.**

1. HCA of 165 HCC NAT samples derived from 33 batches of TMT-11 data after batch correction. The protein expression data were transformed by z score normalization. The samples from the same batch show scattered distributions.
2. PCA of 33 batches of TMT-11 data after batch correction. The samples from different batches show no obvious separation in the first and second principle components.
3. Heatmap of 5999 DEPs among the three tumor subgroups using the criteria of q-value <0.05.


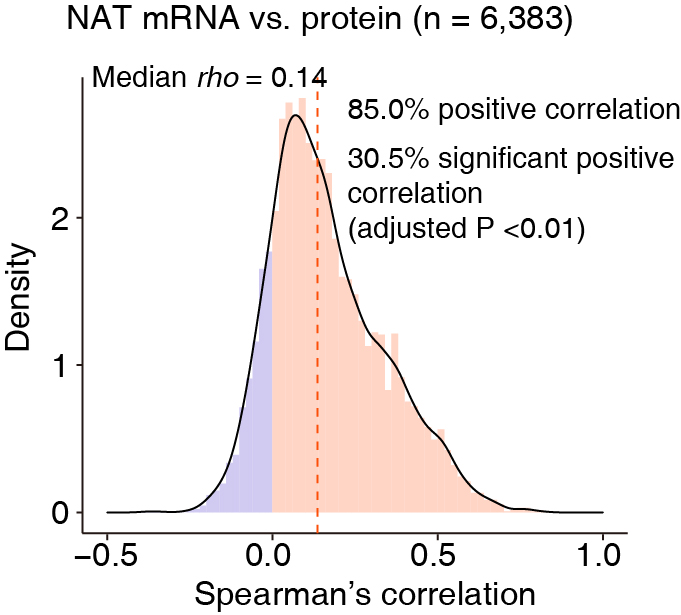


**Figure S2. Distributions of Spearman’s correlation coefficient (rho) between mRNA and protein of NATs at gene level.**

Totally, 85.0% mRNA-protein pairs across the 159 samples were positively correlated, and 30.5% showed significant positive correlation (multiple-test adjusted p < 0.01). The median of *rho* was 0.14 in all mRNA-protein pairs. P-value was calculated by using a Kolmogorov-Smirnov test. Light red bars indicated positive correlations, and light blue ones indicated negative correlations.


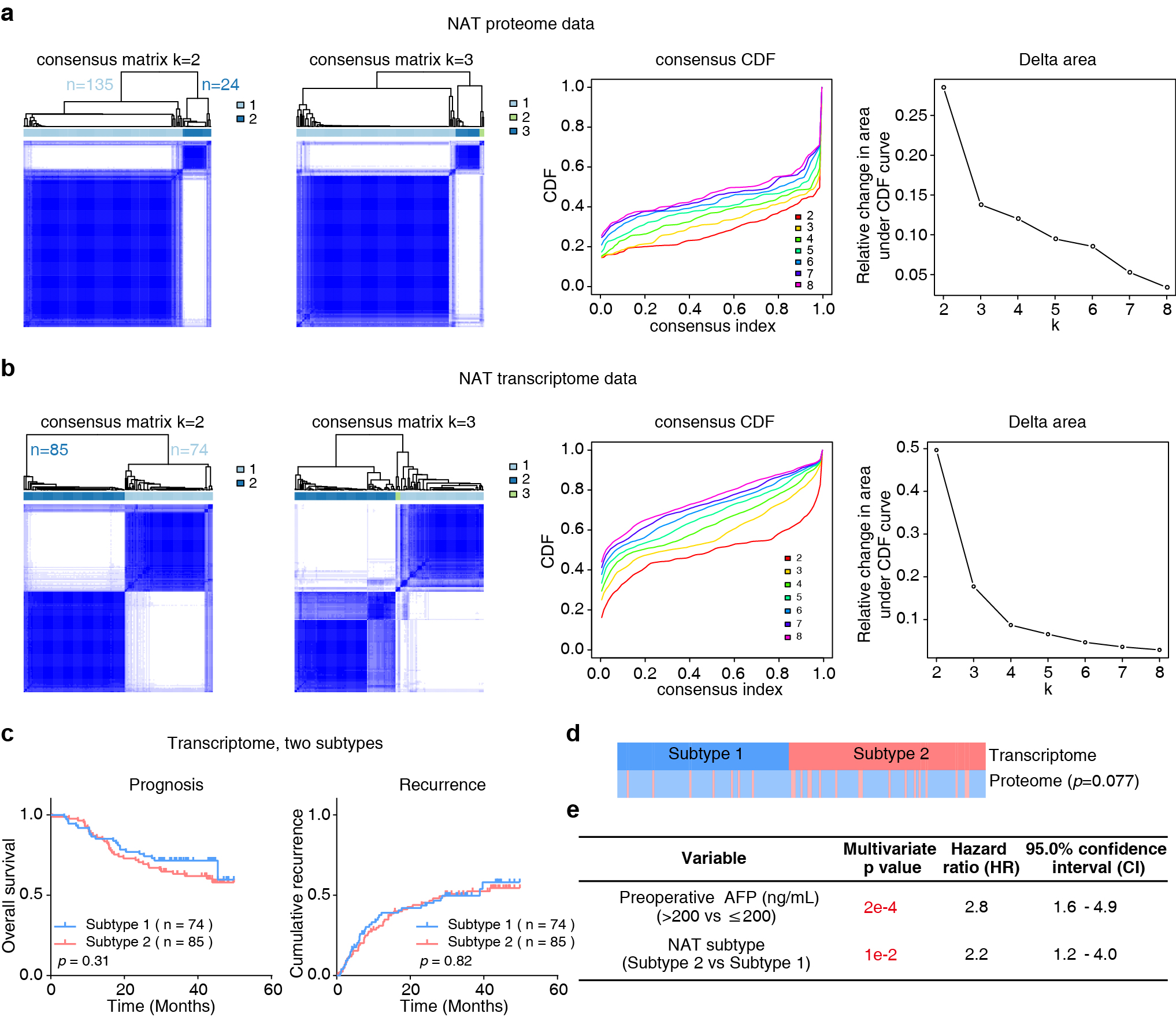


**Figure S3. Consensus clustering using the transcriptome and proteome data.**

1. The top varied 759 proteins (MAD >0.3) of NAT proteome data (~12%) were used to perform K-means consensus clustering to identify subtypes. K was tested from 2 to 8 and consensus clustering was based on 1,000 resampled datasets. The consensus matrix of k=2 and 3, consensus cumulative distribution function (CDF) plot and delta area (change in CDF area) plot were shown.
2. The top varied 2000 genes of NAT transcriptome data (~10%) were used to perform K-means consensus clustering to identify subtypes.
3. Kaplan-Meier curves of overall survival and cumulative recurrence for the two transcriptome subtypes. Subtype 1, n = 74; Subtype 2, n =85. (Log-rank test)
4. Sample comparations between the transcriptome and proteome subtypes. Fisher’s exact test was used to test the consistency between transcriptome and proteome subtypes.
5. Multivariate Cox regression analysis (overall survival) of two proteome subtypes after controlling for AFP.


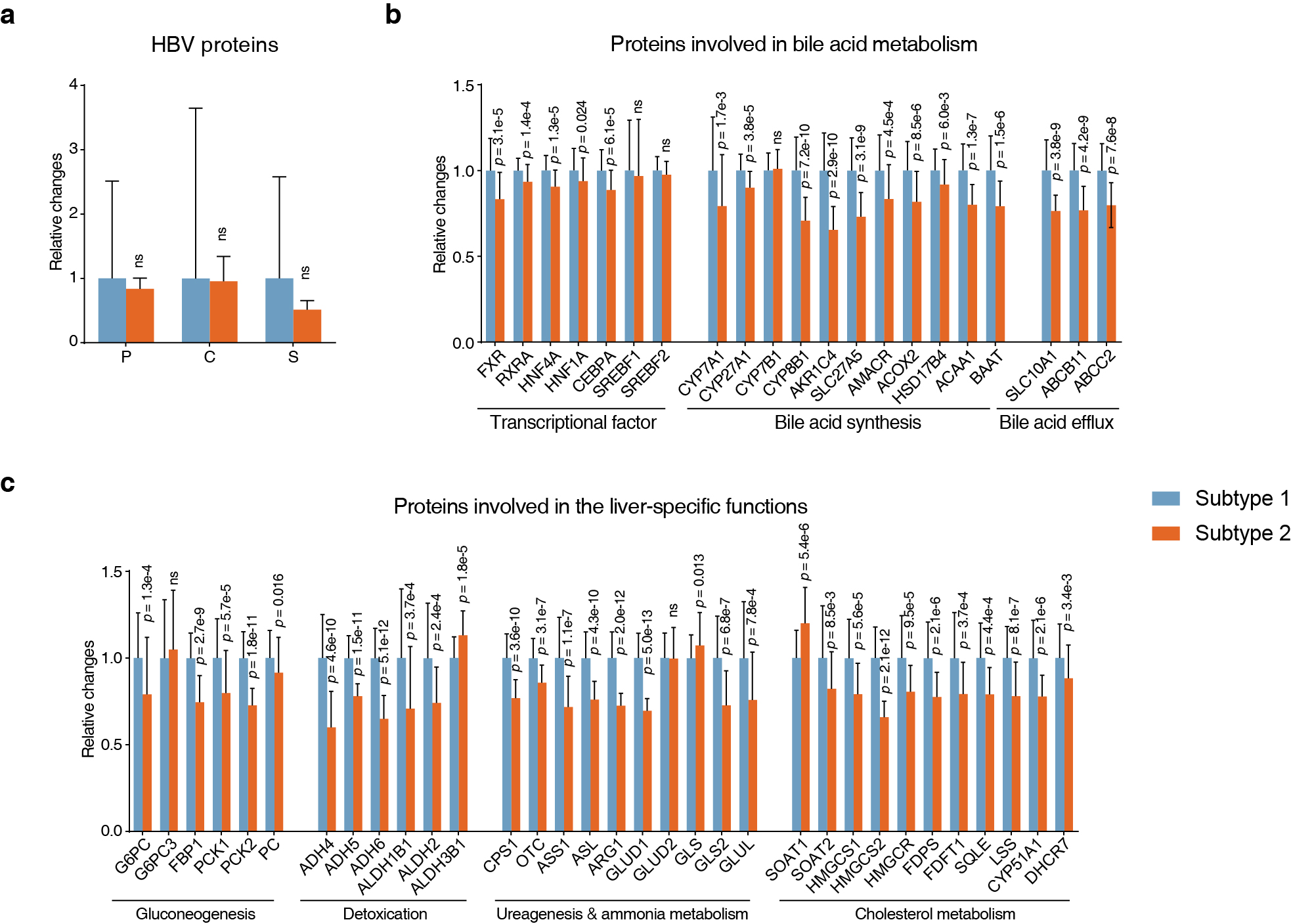


**Figure S4. The proteomic differences of HBV and liver-specific metabolism-related factors.**

1. The expression differences of HBV viral proteins between the two NAT subtypes. (Mann-Whitney test)
2. The expression differences of bile acid metabolism-related proteins between the two NAT subtypes. (Mann-Whitney test)
3. The expression differences of proteins involved in liver-specific function between the two NAT subtypes. (Mann-Whitney test)


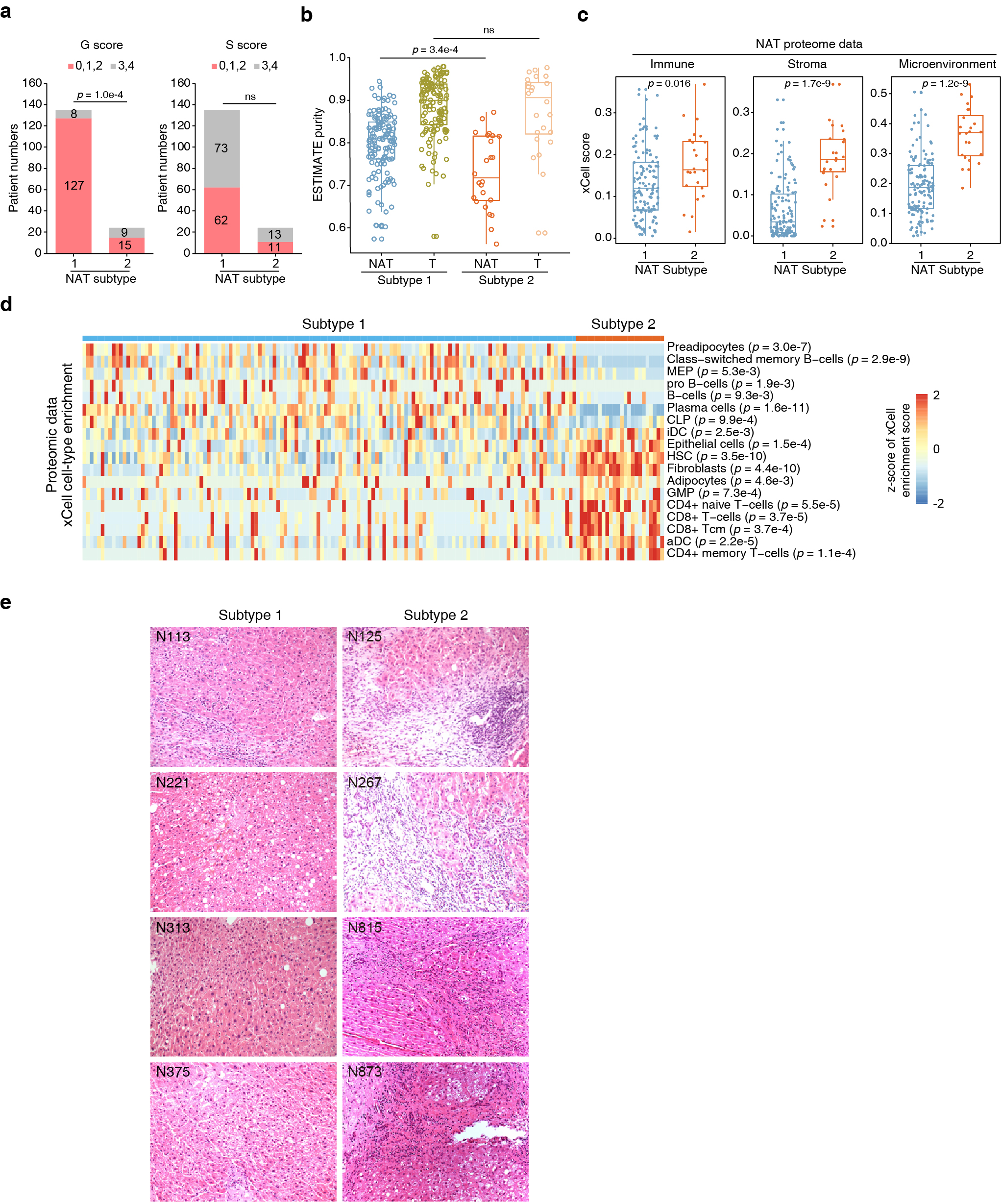


**Figure S5. Immune infiltration analysis using the proteome data.**

1. Association of clinically G scores and S scores with the two NAT subtypes. (Fisher’s exact test)
2. Boxplots showing the purity of the two NAT subtypes and their paired tumors calculated by ESTIMATE algorithm. (Mann-Whitney test)
3. Boxplots showing immune, stroma and microenvironment scores of the two NAT subtypes calculated by xCell using z-score normalized proteome data.
4. Heatmap illustrating significantly differential cell type compositions calculated by xCell between two NAT subtypes using z-score normalized proteome data. (Mann-Whitney test p-value). The xCell cell-type scores were normalized by z-score. MEP, megakaryocyte-erythroid progenitor; CLP, common lymphoid progenitor; HSC, hematopoietic stem cell; GMP, granulocyte-macrophage progenitor.
5. Representative hematoxylin and eosin (H&E) staining of sections of two NAT subtypes. The patient identification number was labeled in the upper left (magnification, ×200).


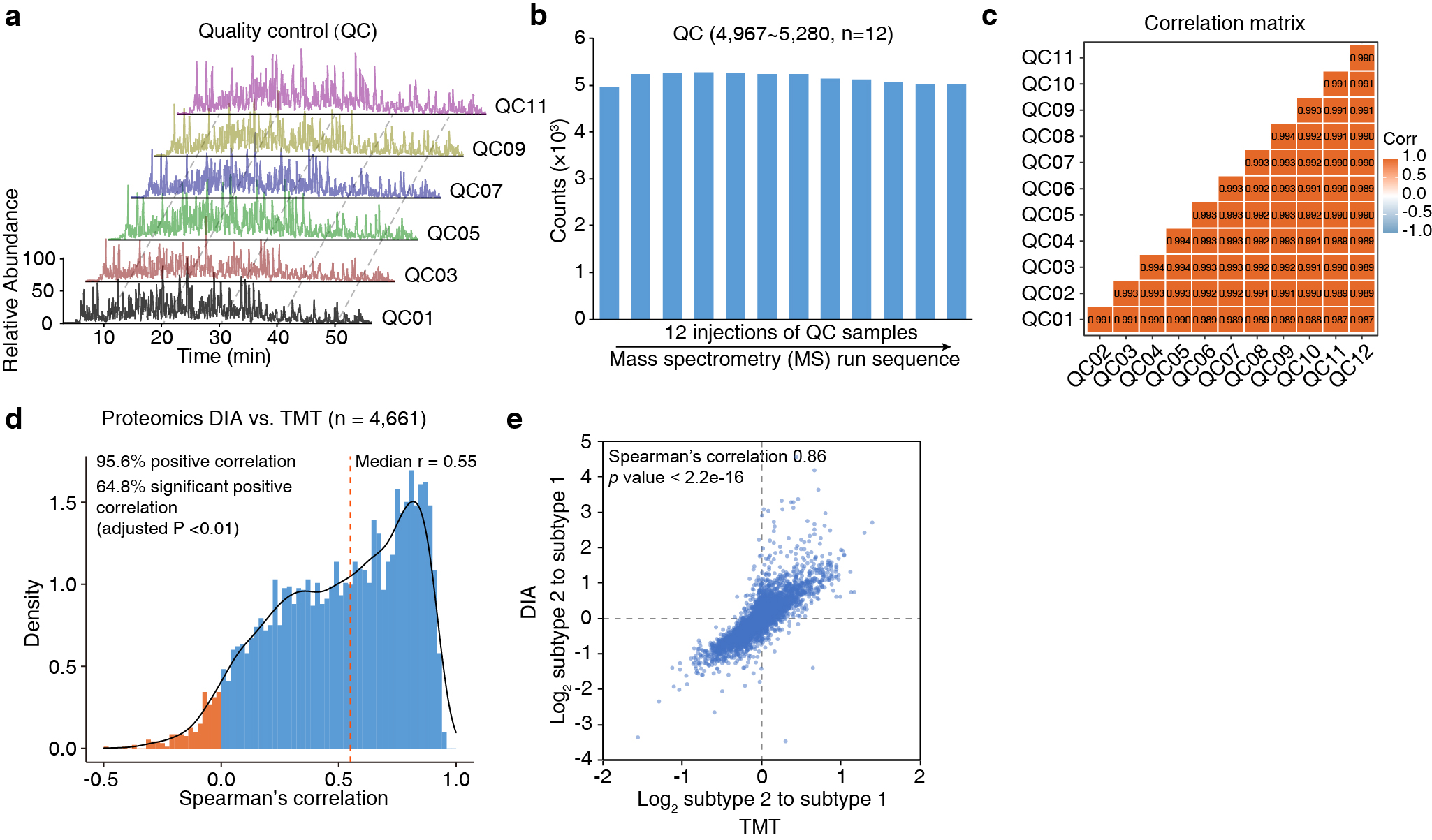


**Figure S6. The data quality control analysis of the data-independent acquisition (DIA) dataset.**

1. The quality control (QC) sample which was a mixture of all the sample peptides was analyzed using the same DIA method as the NAT and tumor samples. Twelve QC samples were analyzed after every ten HCC NAT or tumor samples by mass spectrometry (MS). Base peaks of the selected six injections were aligned to demonstrate the stability of liquid chromatography- mass spectrometry (LC-MS).
2. The barplot showed the protein identification counts of the twelve injections of mix samples.
3. The quantification correlation matrix of the twelve injections of mix samples.
4. The quantification consistency between DIA and tandem mass tags (TMT) data of the 46 NAT samples in protein level (n=4661). Totally, 95.6% proteins across the 46 samples were positively correlated, and 64.8% showed significant positive correlation (multiple-test adjusted p < 0.01). The median of *rho* was 0.55 in all proteins.
5. The Spearman’s correlation of the relative changes of Subtype 2 NATs to Subtype 1 NATs between TMT and DIA data.


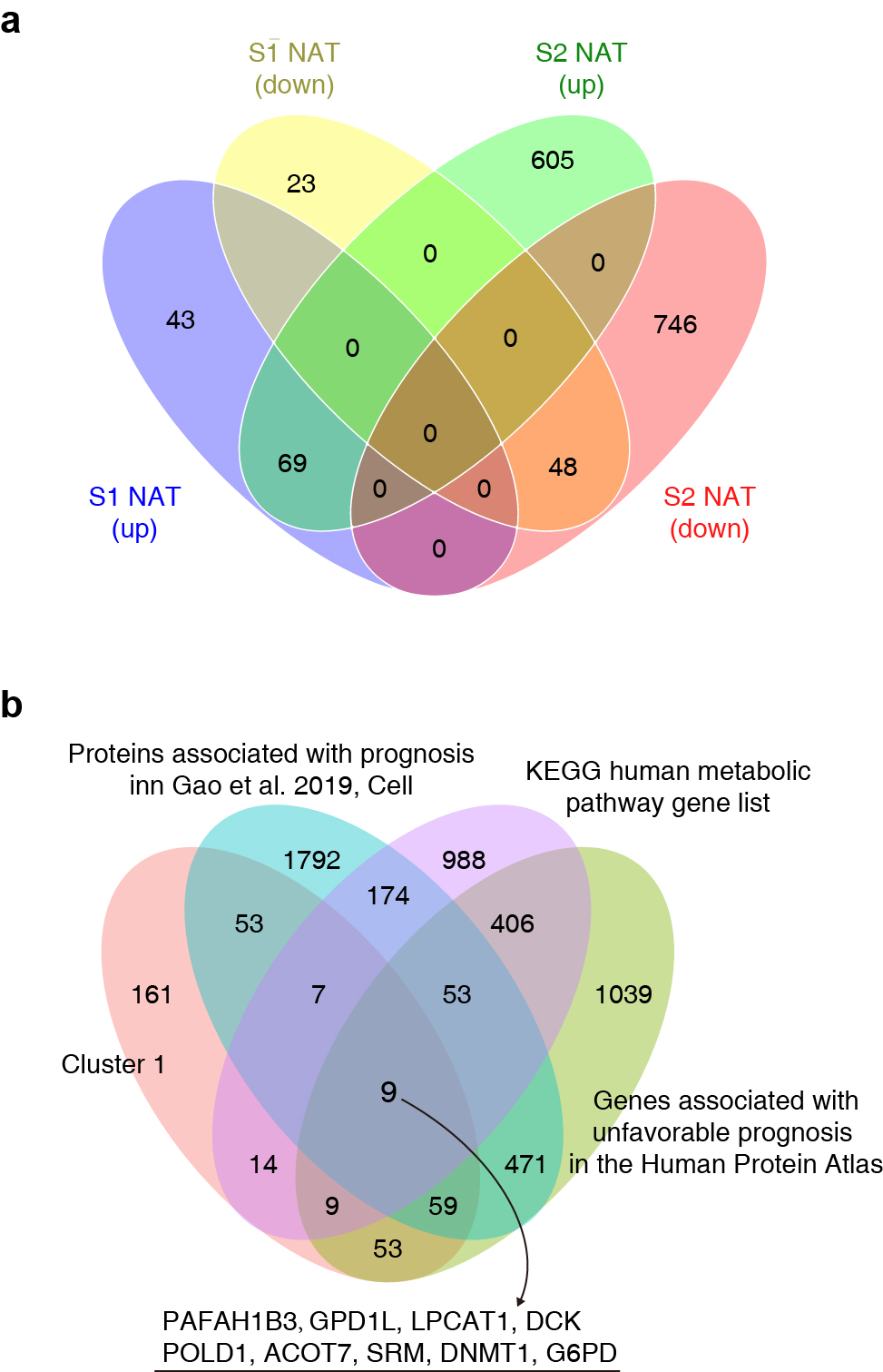


**Figure S7. Overlap analysis.**

1. The overlap analysis of the up-regulated and down-regulated proteins in Subtype 1 NATs and Subtype 2 NATs
2. A 4-dimensional Venn plot shows the overlap of four groups of proteins, 1) Proteins in cluster 1 in Fig. 6b; 2) Genes associated with unfavorable prognosis of HCC in the Human Protein Atlas (https://www.proteinatlas.org/); 3) Proteins associated with HCC prognosis in our previously published paper; 4) Proteins involved in KEGG human metabolic pathway gene list.
